# Pilot Testing and Validation of an Educational Game on Transportation Challenges for Mobility Device Users

**DOI:** 10.3390/disabilities4040051

**Published:** 2024-10-13

**Authors:** Jorge L. Candiotti, Sangmi Park, Chang Dae Lee, Evan J. Rafferty, Rosemarie Cooper, Rory A. Cooper

**Affiliations:** 1 Human Engineering Research Laboratories, Department of Veterans Affairs Pittsburgh Healthcare System, University of Pittsburgh, Pittsburgh, PA 15206, USA; 2 Department of Bioengineering, University of Pittsburgh, Pittsburgh, PA 15213, USA; 3 Department of Rehabilitation Science and Technology, University of Pittsburgh, Pittsburgh, PA 15213, USA; 4 Department of Occupational Therapy, Indiana University Indianapolis, Indianapolis, IN 46202, USA; 5 Department of English, Kenneth P. Dietrich School of Arts and Sciences, University of Pittsburgh, Pittsburgh, PA 15260, USA; 6 Department of Physical Medicine and Rehabilitation, University of Pittsburgh, Pittsburgh, PA 15213, USA

**Keywords:** gamification, education, transportation, accessibility, mobility devices

## Abstract

Despite the increasing use of assistive mobility devices, practical education to navigate real-world ground transportation barriers is lacking. The educational board game, called HERL-Town, was developed to teach safe and effective navigation for mobility device users (MDUs) in the community. The study examined the initial validity, reliability, and overall quality of HERL-Town as an educational tool for overcoming transportation barriers in real-world environments. HERL-Town featured fifty scenarios focused on transportation barriers and strategies, which were assessed for content validity, while the game quality was evaluated using the Model for the Evaluation of Educational Games (MEEGA+) tool. Twenty-three experienced MDUs and four caregivers participated in the study. The results indicated a good quality score of 60.15 and forty-five scenarios met the content validity standards. The overall reliability of the scenarios was moderate (ICC = 0.729). Early psychometric findings suggest HERL-Town as a promising effective educational game for helping new MDUs and their travel companions navigate safe and effective ground transportation barriers, hence enhancing their confidence, independence, and participation in the community.

## 1. Introduction

There are approximately 6.8 million adults who benefit from mobility devices in the US [1,2] and about 606,000 new mobility devices are prescribed annually to newly injured people with limb amputations, spinal cord injuries, and other conditions [3]. Mobility devices are a subcategory of assistive technologies that range from walkers and canes to advanced wheeled technology such as scooters, manual, and power wheelchairs. Newly injured mobility device users (MDUs) typically undergo rehabilitation training to operate mobility devices. However, this training may be brief, inconsistent, and subject to variation based on the therapist’s experience [4]. Additionally, it often prioritizes skill acquisition while potentially overlooking the development of situational awareness needed to navigate community environments and transportation-related barriers. The lack of technical and situational skills, decision-making experience, and inaccessible environments significantly impact the social participation, health, and quality of life of MDUs [5,6]. While web-based applications have been designed for accessible route planning and public transportation assistance [7,8], these tools typically focus on public transit, often overlooking the need for strategic skills to improve the situational awareness and decision-making of MDUs in navigating ground transportation challenges. For instance, people with disabilities have difficulty planning their trip and getting on and off the bus, lack knowledge and experience, and perceive or experience safety concerns [7,9]. Moreover, while accessibility for MDUs has been significantly improved since the passage of the Americans with Disabilities Act (ADA) in 1990, accessibility issues persist [10,11]. Ultimately, environmental and transportation barriers may increase MDUs’ reliance on family or professional caregivers [12,13]. Additionally, inexperienced family caregivers traveling with MDUs may be at increased risk when facing ground transportation barriers. Improving the learning methods about real-world situations that MDUs are likely to experience may enhance their mobility, confidence, and safety in navigating in the community.

Educational board games are considered an effective method with which to enhance knowledge and learning in both children and adults [14–17]. In clinical settings, educational board games can enhance learning and encourage behavioral changes [18] which may increase confidence in MDUs while navigating ground transportation barriers and advocating for their mobility rights in society. A systematic review showed that board games influence educational knowledge in clinical fields with effect sizes ranging from very small to large [19]. Although no serious games currently focus specifically on addressing transportation barriers and strategies for MDUs, it is important to highlight the potential of popular board games (e.g., Monopoly, Ticket to Ride, and Pandemic, among others) to foster strategic and decision-thinking, resource management, situational awareness, and infrastructure planning. In addition to the gameplay and player interaction associated with board games; these benefits can be harnessed to develop serious games with an educational purpose to simulate transportation barriers and decision-making scenarios for MDUs. Given the positive effectiveness shown in this evidence, learning about ground transportation barriers and how to overcome them may improve the community mobility of MDUs and facilitate their social participation and community integration.

The Educational board game, known as “HERL-Town”, was designed to be intuitive, easy to play, entertaining, and educational for learning about ground transportation barriers that MDUs may encounter throughout their travel journey in the community and strategies to overcome them. HERL-Town included active learning and teamwork features [15,16,20–22] to facilitate interaction with other players and exchange travel experiences, particularly if MDUs or travel companions participate in the game. Before introducing HERL-Town to the target audience (i.e., new MDUs, caregivers, travel companions, and rehabilitation professionals), this study aimed to conduct a pilot testing of the gaming quality of HERL-Town and to examine its content validity and reliability with an experienced target audience. Therefore, three criteria were set:

Criterion 1: The gaming quality score for HERL-Town will be higher than 65 points, indicating excellent gaming quality, supported by the Model for the Evaluation of Educational Games (MEEGA+) questionnaire [23];

Criterion 2: The scenario cards, which are the core educational content of HERL-Town, will have acceptable levels of content validity;

Criterion 3: The scenario cards, which are at the core educational content of HERL-Town, will have at least a moderate level of reliability.

## 2. Materials and Methods

### 2.1. Protocol

The protocol was approved by the Veterans Affairs Institutional Review Board (approval number: 1744825), ensuring compliance with ethical standards for research involving human participants. Written consent was obtained from participants. After completing a demographic questionnaire, participants played the HERL-Town game in groups of 3–6 players. After playing HERL-Town, participants completed a questionnaire assessing the gaming experience, validity, and reliability. Providing additional comments and feedback on the game and scenario cards was optional. Participants could complete the questionnaire in person or submit it by mail if they needed more time to complete it.

### 2.2. Participants

Participants included MDUs or travel companions (e.g., caregivers or family members) who assist MDUs with ground transportation. The inclusion criteria for MDUs were as follows: (1) 18 years or older, (2) use a mobility device as the primary means of mobility, and (3) use a mode of ground transportation (e.g., personal, or public transportation) at least once per week. The inclusion criteria for travel companions were as follows: (1) 18 years or older, and (2) traveled with MDUs at least once per week. Recruitment and data collection took place at the National Veterans Wheelchair Games in Portland, Oregon, and at the Human Engineering Research Laboratories in Pittsburgh, Pennsylvania from August to December 2023.

### 2.3. Materials

#### 2.3.1. Creation of Transportation Barrier Scenarios and Answers

HERL-Town was developed at the Human Engineering Research Laboratories (HERL) as an educational resource to address transportation challenges faced by MDUs throughout their journeys. Under a grant from the US Department of Transportation [24], we were challenged to create an education and implementation tool for the results of a study that included a large-scale survey, focus groups, and journey mapping. The goal was to create a tool that could improve knowledge among end-users, travel companions, students in health, planning, and transportation professions, and service providers. The current version of HERL-Town includes 50 scenarios that reflect real-world situations gathered from user experiences, which are used for educational purposes during the game. The scenarios and potential responses were developed by a collaborative working group that included various stakeholders, such as MDUs and their caregivers/travel companions with over 10 years of using manual and power wheelchairs and different modes of transportation (e.g., bus, taxi, paratransit, and personal vehicle), and an interdisciplinary team of rehabilitation experts and assistive technology specialists. This collaborative effort was moderated by a wheelchair user with over 40 years of experience designing and testing assistive technology following a modified Delphi method [25]. First, the interdisciplinary team developed a list of scenarios and potential responses derived from transportation barriers reported by MDUs. These barriers were encountered across various modes of transportation, including private vehicles, public transportation, and paratransit services [26]. Data to create the scenarios were acquired from surveys, focus groups, and a journey-mapping process [27–29]. Two main categories were identified among the reported travel barriers: vehicle-specific and infrastructural. Vehicle-specific barriers included, for example, vehicle ingress/egress and wheelchair securement, while infrastructural barriers included, for example, access to the service location, transportation schedules, and difficulty finding accessible parking. The interdisciplinary team subsequently met with stakeholders to discuss and refine the list of scenarios and responses, incorporating additional suggestions from their experiences encountering transportation barriers. Following this, the team revised the scenarios and held another meeting with the stakeholders to reach a consensus on the final scenarios and responses. Three meetings were conducted and the outcome was a well-rounded agreement, reflecting the collective judgment of both the stakeholders and the interdisciplinary team.

#### 2.3.2. Development of HERL-Town, an Educational Transportation Board Game

**Figure 1.**
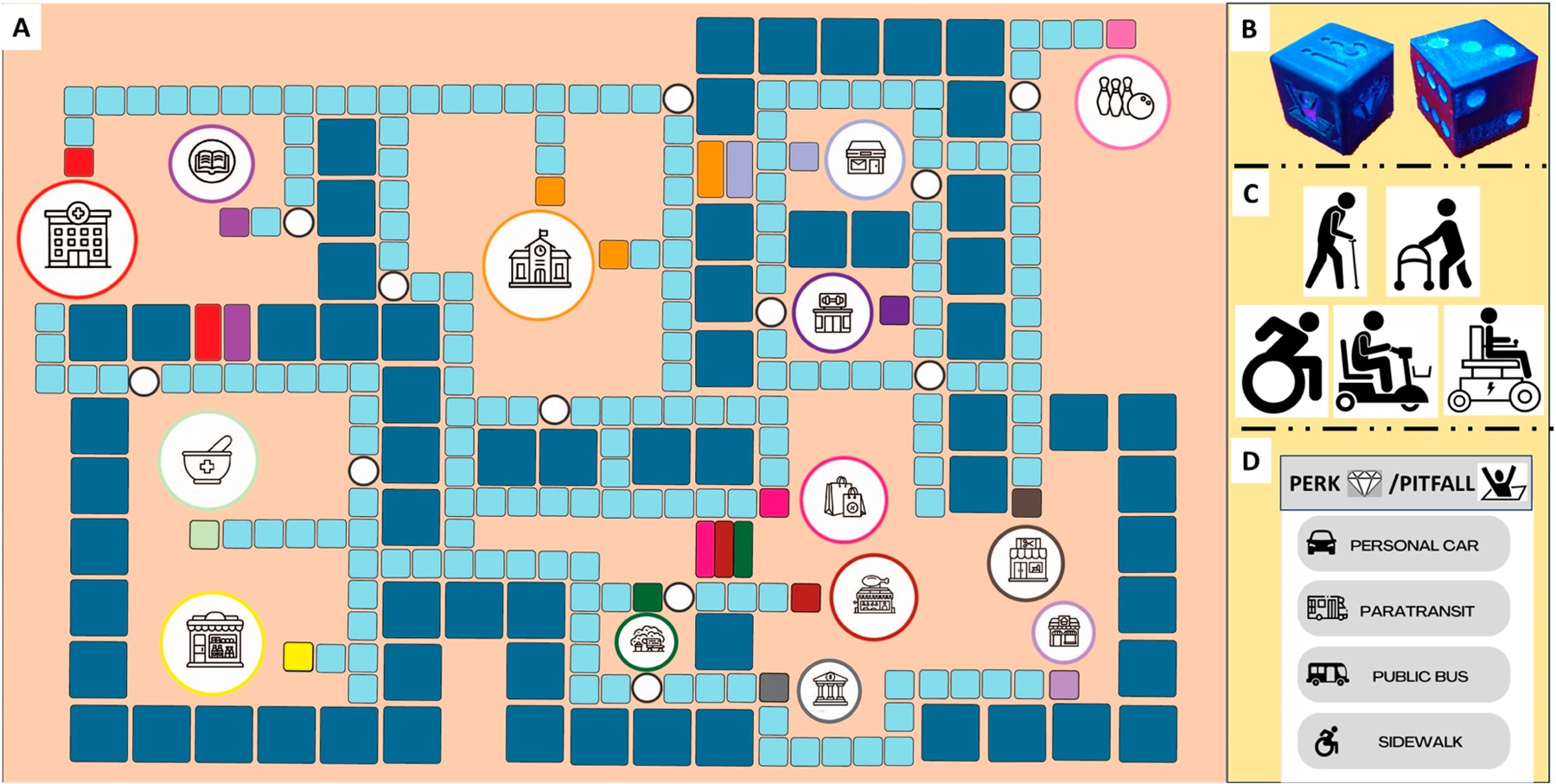
Components of the educational board game. (**A**) Map layout, (**B**) Numbered and event dice, (**C**) Game characters, (**D**) Event cards.

HERL-Town was developed using the Reference Model for Applying Gamification in Education [30], a gamification framework designed to incorporate game design elements in non-game contexts. The iterative design process involved collaboration among stakeholders and the interdisciplinary team. It required a thorough characterization of the context and activities, identification of game objectives, selection of game design elements, analysis of player outcomes, and integration of educational content into the activities (i.e., scenarios and responses). Illustrations of HERL-Town components were presented in Figure 1: (A) A community map consisting of sidewalks (cyan tiles), road (blue tiles), bus stops (white circles), and community facilities; (B) Two dice: A numbered die that determined the number of moves and another die marked with cards that determined trump cards (i.e., perk or pitfall) or educational scenario card; (C) Five types of pawns representing a mobility device (i.e., cane, walker, scooter, and manual or power wheelchair). The mobility device did not have a significant role in the game; (D) Trump cards included perk, pitfall, or scenario cards. Players may receive a reward or penalty based on the chosen mode of transportation if they roll a perk or pitfall die, respectively. The mode of transportation included walking on the sidewalk, public vehicle, paratransit/taxi, or private vehicle; the scenario card included a transportation barrier situation with multiple-choice responses obtained from meetings with stakeholders and the interdisciplinary team; and (E) In-game money ranging from $1, $5, $10, and $20 (not included in Figure 1). All scenario cards and responses were presented in Supplementary Table S1.

A research team member acted as the moderator, managing the game, handling the money, explaining the rules, and assisting with the movement of players’ pawns. At the beginning of the game, the players receive the same amount (i.e., $15) of in-game money, which they use for fares or fines during the travel journey within the game. All players have the same starting points and destination, which were selected by the players at the beginning of the game.

In each turn, players roll a numbered die and another die to receive a trump card including a scenario, pitfall, or perk card. According to the outcome of the numbered die, players moved using the sidewalk tiles or road tiles. Moving on the road tiles meant the player was using a mode of transportation (i.e., personal vehicle, paratransit, or public vehicle). Each mode of transportation moved on a unique number of tiles in the game, mirroring its availability and real-life transportation capabilities corresponding to its fare in the game. For example, players who chose a personal vehicle as their transportation method moved faster with greater flexibility. Compared to paratransit or bus users, who must wait one turn representing reservation time for paratransit or waiting for the bus, personal vehicle users could move immediately. Hence, players may travel faster using the road (i.e., bigger tiles) than moving on the sidewalk (i.e., smaller tiles). Perk cards represented travel facilitators, such as cash bonuses and extra moves, while pitfall cards indicated travel barriers and challenges, with penalties, encountered by MDUs in communities. A scenario card consisted of a real-life ground transportation barrier experienced by MDUs with multiple-choice options that are potential solutions to overcome the given scenario. Players who selected the most suitable response received additional in-game rewards, such as extra moves or game currency, enhancing their progress. The game was designed for up to six players/teams and typically lasts about an hour.

### 2.4. Metrics

#### 2.4.1. Model for the Evaluation of Educational Games (MEEGA+) Questionnaire

The MEEGA+ [23,31] was employed to evaluate the gaming experience with HERL-Town. The MEEGA+ was a revised version of the MEEGA questionnaire developed to assess the gaming quality of educational games [32]. The MEEGA+ consisted of 35 items categorized into nine dimensions: usability, confidence, challenge, satisfaction, social interaction, fun, focused attention, relevance, and perceived learning. The usability dimension comprised five sub-dimensions: aesthetics, learnability, operability, accessibility, and user prevention/recovery. Cronbach’s alpha of overall items of MEEGA+ showed a high reliability of 0.927 [23] and an excellent internal consistency of 0.970.

Participants were asked to respond to each item on a 5-point Likert scale. The quality score was calculated based on the formula provided by the MEEGA+ developers to evaluate the quality of games [31]. Quality scores under 42.5 were classified as low quality, scores between 42.5 and 65 were classified as good quality, and scores higher than 65 were classified as excellent quality.

#### 2.4.2. Questionnaire for the Validity and Reliability of the Educational Content of the ETBG

##### a. Content Validity of Scenario Cards

HERL-Town included 50 scenario cards used for educational purposes during the game. To examine their content validity, participants were asked to rate agreement on the question ‘Do you agree that the situation is realistic?’ using the 5-point Likert scale (1: strongly disagree, 2: disagree, 3: neutral, 4: agree, and 5: strongly agree).

##### b. Inter-Rater Reliability of Scenario Cards

Each scenario card included four responses that encompassed MDU problem-solving strategies. These responses included a mixture of safety-focused, convenience-focused, and active-passive strategies. To examine inter-rater reliability, participants were asked to choose one best response to each scenario question ‘What do you do in this situation?’.

### 2.5. Statistical Analysis

Descriptive analyses were conducted to analyze the demographic characteristics of the participants. Bar graphs of the MEEGA+ items were used to assess user experience including usability dimension and learning experience. Furthermore, the original 5-point Likert scale was coded into three categories for better data visualization (i.e., 1 and 2 represented ‘disagree, 3 for ‘indifferent’, and 4 and 5 represented ‘agree’).

Item-level content validity indices (I-CVIs) and Content Validity Ratio (CVR) were calculated to examine the content validity of 50 scenario cards. The sum of respondents who answered 5 (i.e., strongly agree) and 4 (i.e., agree) was divided by the total number of respondents to calculate I-CVIs. The CVRs were calculated by using the original formula [33]. Missing values were excluded from calculations for I-CVIs and CVRs. Scenario cards with I-CVI scores of 0.78 or higher [34] and CVRs satisfying the critical level of agreement according to the number of respondents [35] were considered to have an acceptable level of content validity.

The Intraclass Correlation Coefficient (ICC) was analyzed to examine the inter-rater reliability. For this study, an ICC between 0.5 and 0.75 [36] was considered to have a moderate level of reliability. Additionally, the frequency of selected responses to the question ‘What do you do in this situation?’ for each scenario card was counted to understand the most appropriate response from the MDUs’ perspectives among the four responses. Microsoft Excel was used to calculate I-CVIs and CVRs, and SPSS 29 (IBM Corp., Armonk, NY, USA) was used to analyze ICC scores.

## 3. Results

A total of 27 participants (18 male and 9 female) completed the study. The sample size satisfied the recommended number of participants for a pilot content validity test (i.e., 15 to 30 persons) [37]. Participants included four caregivers/travel companions and 23 MDUs (manual wheelchair users: 10, power wheelchair users: 3, scooter users: 2, ambulatory assistive device users: 3, and lower prosthetic users: 3). The mean age of participants was 59.4 *±* 15.1 years. More than half of the participants were retired (*n* = 15). The remainder of the participants included six full-time employees, one part-time employee, one self-employed person, one student, and three currently unemployed people. Regarding residential settings, 10 participants reported living in urban areas, 13 in suburban areas, and 1 in rural areas.

### 3.1. Criterion 1: Game Quality Test

**Figure 2.**
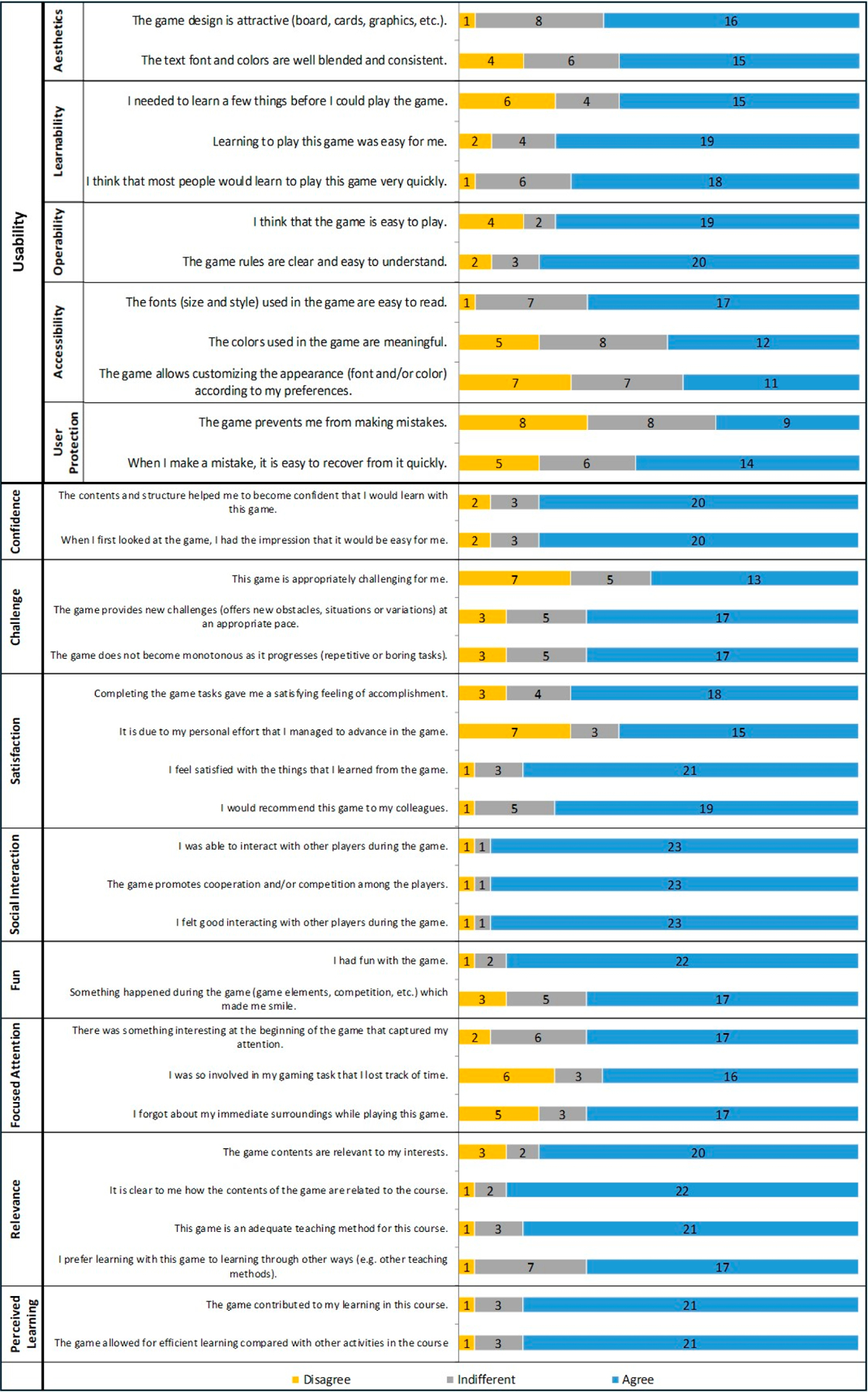
Results across the nine dimensions of MEEGA+ for the ETBG HERL-Town. The original 5-Likert scale was collapsed into three categories: ‘Disagree’ (strongly disagree and disagree), ‘Indifferent’ (neither agree nor disagree), ‘Agree’ (agree and strongly agree).

Data from 25 participants that did not have missing values were used for analysis. Participants reported the gaming quality of HERL-Town to be 60.15. Although the score did not meet criterion 1 (i.e., excellent quality; scores higher than 65), the results indicated good quality. The visualized results across the MEEGA+ nine dimensions were presented in Figure 2. Among the 35 items of MEEGA+, the agreement rate for four items was under 50% including the usability dimension (‘The color used in the game are meaningful’, ‘The game allows customizing the appearance according to my preferences’, and ‘The game prevents me from making mistakes’) and one item in the challenge dimension (‘This game is appropriately challenging for me’).

### 3.2. Criterion 2: Content Validity of the Scenario Cards

Due to missing data within individual respondents, data from 24 out of 27 participants were used to analyze the content validity. The CVR and I-CVI values to examine the content validity of 50 scenario cards included in HERL-Town were presented in Table 1. Five scenario cards did not meet the standards for an acceptable level of content validity. Scenario card #8 did not satisfy both the CVR and I-CVI standards. Four scenario cards (#2, #18, #19, and #20) satisfied the CVR standards but did not meet I-CVI’s. These results led to the partial acceptance of Criterion 2.

### 3.3. Criterion 3: Reliability of the Scenario Cards

Data from 16 participants that did not have errors (e.g., missing values, and multiple answers) were used to analyze the inter-rater reliability. The ICC for overall scenarios was 0.729 (95% Confidence Interval: 0.609; 0.826). The ICC, indicating a moderate level of reliability [36], led to the acceptance of Criterion 3. The most common responses for each scenario card were highlighted in bold in Table S1.

## 4. Discussion

The results of this study demonstrated that the HERL-Town board game exhibited good gaming quality and initial evidence of validity and reliability were established with community-dwelling MDUs and caregivers/travel companions. These results supported the educational potential of HERL-Town as a gamified training tool addressing safe and effective navigation when encountering ground transportation barriers.

Among the three quality criteria according to the MEEGA+ (i.e., low, good, and excellent), the gaming quality score of HERL-Town indicated a good level of quality. This level of gaming quality was interpreted as moderate levels of social interaction, fun, and attention; however, it may need improvement regarding rule clarity and ease of play [23]. For instance, the usability of the board game showed mixed results, particularly in the sub-dimensions of user protection and learnability. The rules were complicated and unclear, leading to participants making mistakes despite multiple reminders during the game. Clear and concise rules are essential to minimize errors and facilitate swift recovery from mistakes. Participants reported discrepancies across various items within the usability dimension. Aspects such as aesthetics and accessibility hold significant importance when engaging with MDUs who may not only experience limitations in mobility but also be visually impaired.

The components of HERL-Town, including the map, dice, game currency, and various cards, were specifically designed to serve educational objectives, integrating real-life scenarios for MDUs [14,16–18]. This design was informed by the knowledge and experience of a diverse group of stakeholders, including rehabilitation professionals, engineers, experienced MDUs, and caregivers or travel companions. The high rate of agreement on the perceived learning dimension and acceptable levels of content validity found in this study supported its educational function for MDUs and caregivers/travel companions. To leverage these advantages, future studies should explore the application of HERL-Town for health professionals and students for educational purposes.

To enhance the gaming quality, the revision process should prioritize improving the satisfaction, social interaction, fun, and focused attention dimensions, which are the four features of excellent quality [23], in addition to addressing the dimensions with lower scores in this study. Among the four features to be improved, the scores of the focused attention dimension were lower than the other three dimensions in the current study. As the current version is a multiplayer game, waiting for their next turn may be a distracting factor for players. A revision to the rules to maintain all players’ concentration may be needed, for example, providing open opportunities to correct another player’s response when it is deemed insufficient or inadequate. The revisions could also include adding elements such as special pitfall cards (e.g., bus breakdown and adverse weather) that can be used during another player’s turn to influence their actions. In addition, developing a digital version of the game, tailored to the player’s mobility method, could reinforce focused attention. Digital games offer greater customization for learners to control their learning path, and include game elements not typically available in physical board games (e.g., music, information pop-ups, hints, etc.), making the game easier to use, increasing attention and knowledge retention [38]. Additionally, accompanying this digitalization with revised scenarios featuring a broader range of situations may address the low rate of agreement on the usability and challenge dimensions identified in the study.

In this study, Scenarios 2 (S2), 8 (S8), 18 (S18), 19 (S19), and 20 (S20) did not meet the standards for I-CVI and CVR. The CVRs for S2, S19, and S20 were found to be marginally short of the standard. Overall, the content validity of some scenarios may have been impacted by the ambiguity and long description of the scenarios which might be needed to offer more context to the question but could be improved to reduce boredom and frustration. Retaining S19 and S20 is appropriate considering the gaps between perceived wheelchair skills and objective wheelchair skill performance [39]. As wheelchair users typically utilize paratransit services based on prior reservations, which specify pick-up and drop-off points, the likelihood of S8 (“The paratransit driver stops the vehicle a few hundred feet from your destination and asks you to get off here instead of in front of your destination”) occurring may be low. However, S8 can occur due to paratransit drivers’ inappropriate behavior or inexperience which was pointed out as one concern by paratransit users with mobility disabilities [40]. Constant needs for curb-to-curb service among paratransit users [41] also support keeping S8.

The CVR for S18 also did not meet the standard. S18 asked “You need to change the engine oil of your modified vehicle for wheelchair accessibility. However, car manufacturers refuse to change the oil because of the modification. What would you do?”; participants’ low concern for the scenario may be linked to the lower CVR. The S18 could be seen as unrealistic because changing the engine oil might be straightforward when performed by a mechanic expert. Moreover, considering the advancement of technologies for wheelchair users to use electric or automated vehicles [42–44], expanding the scenarios to encompass electric and automated vehicle users may be appropriate.

Participants also offered additional comments regarding scenarios. While most scenarios represented real-world transportation barriers, participants also suggested creating additional scenarios that depict MDUs navigating within business environments that may or may not be compliant with ADA guidelines. For example, expanding the scenarios into special events (e.g., barriers encountered during business trips) that may not occur every day might assist players in considering other environments in which MDUs may have to alter their day-to-day expectations based on limited accessibility. By adding these uncommon event scenarios, the game could continue to push forward disability education by asking players to imagine themselves in these scenarios.

### Limitations

Due to some limitations, the findings of this study should be carefully interpreted. The MEEGA+ may partially capture features of the current version of HERL-Town because the assessment tool was originally designed for digital-based games. Moreover, missing participants’ responses were excluded from analysis which may contribute to biased results of the study. As we did not have a consensus method for judging whether the missing pattern is systematic or random, further study including a larger sample size is recommended to ensure the reliability of the HERL-Town scenarios. Additionally, most participants were wheelchair users and, while these MDUs often present more transportation barriers, scenario cards should include other populations with limited mobility. Lastly, the real-world situations presented in the scenarios may be constrained by geographic and social factors that influenced the research team in developing the transportation barriers and solutions. While MDUs validated most scenarios from across the US, further studies should include a larger and more diverse sample size, extending the game’s scenarios to reflect transportation challenges from various geographic regions across the US.

## 5. Conclusions

The study introduced HERL-Town, an educational board game to teach community-dwelling MDUs safety and effective navigation when facing ground transportation barriers. The findings demonstrated HERL-Town as a promising educational tool for new MDUs, caregivers/travel companions, and rehabilitation professionals. The study showed that a user evaluation of HERL-Town with a larger population should be considered to reinforce the game quality and its educational impact on new MDUs to enhance their mobility, confidence, and independence in real-world environments.

## Supplementary Material

Table S1

## Figures and Tables

**Table 1.** Content validity examination of 50 scenario cards in HERL-Town. I-CVI = Item Level Content Validity Index, CVR = Content Validity Ratio.

Scenario Number	Experts Agreed to Be Realistic or Very Realistic	Experts Agreed to Be Neutral, Unrealistic, or Very Unrealistic	Experts Unanswered	CVR	I-CVI
1	23 (85.19%)	3 (11.11%)	1 (3.70%)	0.77	0.88
2	20 (74.07%)	6 (22.22%)	1 (3.70%)	0.54	*0.77*
3	22 (81.48%)	3 (11.11%)	2 (7.41%)	0.76	0.88
4	22 (81.48%)	3 (11.11%)	2 (7.41%)	0.76	0.88
5	20 (74.07%)	4 (14.81%)	3 (11.11%)	0.67	0.83
6	24 (88.89%)	2 (7.41%)	1 (3.70%)	0.85	0.92
7	23 (85.19%)	2 (7.41%)	2 (7.41%)	0.84	0.92
8	17 (62.96%)	9 (33.33%)	1 (3.70%)	*0.31* ^a^	*0.65*
9	23 (85.19%)	2 (7.41%)	2 (7.41%)	0.84	0.92
10	22 (81.48%)	4 (14.81%)	1 (3.70%)	0.69	0.85
11	20 (74.07%)	5 (18.52%)	2 (7.41%)	0.60	0.80
12	25 (92.59%)	1 (3.70%)	1 (3.70%)	0.92	0.96
13	26 (96.30%)	0 (0%)	1 (3.70%)	1.00	1.00
14	22 (81.48%)	5 (18.52%)	0 (0%)	0.63	0.81
15	25 (92.59%)	1 (3.70%)	1 (3.70%)	0.92	0.96
16	21 (77.78%)	4 (14.81%)	2 (7.41%)	0.68	0.84
17	24 (88.89%)	2 (7.41%)	1 (3.70%)	0.85	0.92
18	19 (70.37%)	8 (29.63%)	0 (0%)	0.41	*0.70*
19	20 (74.07%)	6 (22.22%)	1 (3.70%)	0.54	*0.77*
20	20 (74.07%)	6 (22.22%)	1 (3.70%)	0.54	*0.77*
21	20 (74.07%)	5 (18.52%)	2 (7.41%)	0.60	0.80
22	24 (88.89%)	2 (7.41%)	1 (3.70%)	0.85	0.92
23	22 (81.48%)	5 (18.52%)	0 (0%)	0.63	0.81
24	23 (85.19%)	2 (7.41%)	2 (7.41%)	0.84	0.92
25	21 (77.78%)	5 (18.52%)	1 (3.70%)	0.62	0.81
26	23 (85.19%)	3 (11.11%)	1 (3.70%)	0.77	0.88
27	22 (81.48%)	4 (14.81%)	1 (3.70%)	0.69	0.85
28	23 (85.19%)	3 (11.11%)	1 (3.70%)	0.77	0.88
29	23 (85.19%)	3 (11.11%)	1 (3.70%)	0.77	0.88
30	22 (81.48%)	4 (14.81%)	1 (3.70%)	0.69	0.85
31	20 (74.07%)	5 (18.52%)	2 (7.41%)	0.60	0.80
32	22 (81.48%)	3 (11.11%)	2 (7.41%)	0.76	0.88
33	23 (85.19%)	3 (11.11%)	1 (3.70%)	0.77	0.88
34	23 (85.19%)	3 (11.11%)	1 (3.70%)	0.77	0.88
35	24 (88.89%)	2 (7.41%)	1 (3.70%)	0.85	0.92
36	23 (85.19%)	3 (11.11%)	1 (3.70%)	0.77	0.88
37	21 (77.78%)	4 (14.81%)	2 (7.41%)	0.68	0.84
38	25 (92.59%)	1 (3.70%)	1 (3.70%)	0.92	0.96
39	26 (96.30%)	0 (0%)	1 (3.70%)	1.00	1.00
40	25 (92.59%)	1 (3.70%)	1 (3.70%)	0.92	0.96
41	23 (85.19%)	3 (11.11%)	1 (3.70%)	0.77	0.88
42	23 (85.19%)	2 (7.41%)	2 (7.41%)	0.84	0.92
43	23 (85.19%)	3 (11.11%)	1 (3.70%)	0.77	0.88
44	24 (88.89%)	1 (3.70%)	2 (7.41%)	0.92	0.96
45	24 (88.89%)	1 (3.70%)	2 (7.41%)	0.92	0.96
46	23 (85.19%)	2 (7.41%)	2 (7.41%)	0.84	0.92
47	21 (77.78%)	4 (14.81%)	2 (7.41%)	0.68	0.84
48	20 (74.07%)	5 (18.52%)	2 (7.41%)	0.60	0.80
49	21 (77.78%)	5 (18.52%)	1 (3.70%)	0.62	0.81
50	23 (85.19%)	1 (3.70%)	3 (11.11%)	0.92	0.96

a CVR critical according to panel size: 0.417 (*n* = 24), 0.440 (*n* = 25), 0.385 (*n* = 26), and 0.407 (*n* = 27). Notes: Number of respondents (percentage) was presented. Missing values were excluded from calculations for CVR and I-CVI. CVRs lower than CVR critical and I-CVIs lower than 0.78 were marked in italics.

## Data Availability

The data presented in this study are available upon request from the corresponding author.
