# Supplementary material for: Pilot Testing and Validation of an Educational Game on Transportation Challenges for Mobility Device Users
Source: Disabilities (Basel). Author manuscript; Available in PMC 2025 Jan 2. (PMC11694653; doi:10.3390/disabilities4040051)
Supplement: Table S1 [file NIHMS2035542-supplement-Table_S1.pdf]

Table S1. Fifty scenarios and related responses of the Educational Board Game.

Scenarios
1. You are a power wheelchair user and are parked in an accessible parking space at the mall, but a driver parks in the striped lines next to an accessible parking spot and you cannot get your ramp out. What do you do?<br>**A. Take down the license plate and type of car and bring it into the mall security team so they can contact the owner to move the car.**<br>B. Ask a passerby to move your car a few feet back so that you can get into the car.<br>C. Call a paratransit to come and pick you up from the mall and you can get your car later.<br>D. Call 911 to get assistance with getting into your vehicle.
2. You are a manual wheelchair user and while transferring into your car, one of the wheels from your dismantled wheelchair rolls away and falls out of reach. What do you do?<br>**A. Drive it to the location where your wheel fell and scoop it up from there.**<br>B. Hope that someone else saw what happened and comes by to offer their assistance.<br>C. Call a friend or family member for help.<br>D. Leave the wheel behind and submit an insurance claim when you get home to get a new wheel.
3. You are a power wheelchair user. You get off the bus and realize that the curb cut is blocked by construction. How do you handle finding a way to get around it?<br>A. Try to jump down the curb with your wheelchair.<br>B. Ask a stranger for help.<br>C. Call a family member or friend for help.<br>**D. Wait until the next bus arrives at the station and ask the bus driver for assistance.**
4. You are a manualwheelchair user. You plan to take a bus to get to your destination. When the bus arrives, the ramp is not functioning. What do you do?<br>**A. Wait for the next bus.**<br>B. The bus floor is not too high off the ground. Ask the driver and other passengers to lift you and your wheelchair onto the bus.<br>C. Call the bus company for help.<br>D. Argue with the driver that the ramp is actually working.
5. You are a manual wheelchair user. You get on the bus, but no one helps secure your chair and you start to roll around. What do you do?<br>A. Hold onto the bars tightly and hope that you will not roll around.<br>**B. Ask the bus driver to secure the wheelchair.**<br>C. Ask a nearby passenger for help securing the wheelchair.<br>D. Report this issue to the Bus company.
6. You are a manual wheelchair user. You are waiting at the bus stop when it begins to rain but there is no shelter at the bus stop. What do you do?<br>**A. Find the nearest shelter and keep an eye out for your bus to come.**<br>B. Ask a stranger to shelter you with their umbrella so you can wait in a visible area for the bus driver to see you.<br>C. Ask your friends or family to give you a lift and abandon your bus route.<br>D. Wait in the rain for your bus and hope it comes early.

7. You are a manual wheelchair user, and you are waiting for your paratransit vehicle to arrive. 30 minutes have passed since the scheduled pick-up time with no sign of the vehicle. What do you do?<br>**A. Call the paratransit service and ask for an eta or a new driver.**<br>B. Continue to just wait for the vehicle.<br>C. Call a friend or family member and hope they can give you a ride.<br>D. Call the paratransit service and report your experience and/or concerns
8. You are a manual wheelchair user riding in a paratransit vehicle. The driver stops the vehicle a few hundred feet from your destination and asks you to get off here instead of in front of your destination. What do you do?<br>A. Get off the paratransit vehicle and make your way across the area to your destination.<br>**B. Ask the driver to pull up closer to your destination.**<br>C. Get off the paratransit vehicle and ask a stranger passing by to wheel you to your destination.<br>D. Submit a complaint to the paratransit company.
9. You are a power wheelchair user. You are picked up at 3:00 pm by paratransit for a doctor's appointment at 4:00pm. The driver has many other passengers, and lets you know that you will not be dropped off in time. What do you do?<br>**A. Call your doctor's office to let them know that you will be late and hope that they don't cancel your appointment.**<br>B. Stay on the paratransit and do nothing but risk showing up late to your appointment.<br>C. Call to reschedule your appointment.<br>D. Have the driver drop you off immediately and make your way to the doctor's office on the sidewalk.
10. You are a power wheelchair user. Your wheelchair breaks down in the middle of the street while you are crossing. What do you do?<br>A. Do nothing.<br>**B. Ask nearby people for help.**<br>C. Call the manufacturer/supplier frantically.<br>D. Call 911.
11. You are a manual wheelchair user and encounter residential trash cans in the middle of the sidewalk. You are unable to move them yourself. What do you do?<br>**A. Go back and find another way around.**<br>B. Wait for a passerby and ask them to help move the trash cans.<br>C. Call the residential trash pickup service.<br>D. Knock on the front door of the residence to ask for assistance.
12. You are a power wheelchair user. You are on a sidewalk and encounter vehicles that are parked taking up the entire sidewalk. What do you do?<br>**A. Go back and find another way around.**<br>B. Call a tow truck to move the vehicle out of the way.<br>C. Call the owner and ask them to move the vehicle.<br>D. Call the police for assistance in getting the vehicle moved
13. You are a manual wheelchair user, and you encounter a pothole across the sidewalk that is filled in with gravel. There is a possibility of you getting stuck. What do you do?<br>**A. Go back and cross the street to use the sidewalk on the other side.**<br>B. Ask a passerby to help push you across the uneven sidewalk area.<br>C. Call 911.<br>D. Roll as fast as you can over the sidewalk area and hope that you do not get stuck in the pothole.

14. You are a power wheelchair user. To access the bus station, you must use the elevator, but the elevator is out of service, and the only option to access the bus is by using the stairs. What do you do?<br>**A. Travel to the closest accessible station.**<br>B. Find an employee of the bus company and ask for help.<br>C. You call the management for the public transit to receive further guidance.<br>D. Just give up and go back home.
15. You are a manual wheelchair user. You arrive at your destination and there is a step to get inside the business. What do you do?<br>A. Attempt to get up the step and hope you don't fall.<br>B. Ask nearby people for help.<br>**C. Call the business and ask the manager if they have a ramp or accessible entrance.**<br>D. Give up and go home.
16. You are a manual wheelchair user but ambulatory for a short distance. You just arrived at the grocery store but discover that they do not have a ramp. The store itself is a few steps up from the street level. What do you do?<br>A. Get up and carry your wheelchair up the stairs.<br>B. Ask a stranger to assist you with getting up the stairs.<br>**C. Call the store manager and have a store associate assist you.**<br>D. Turn around and take your business elsewhere.
17. You are a power wheelchair user with an accessible vehicle. The vehicle breaks down and needs to be towed. You can't ride in the tow truck and need to find a way home. What do you do?<br>A. Call an accessible taxi.<br>B. Ask the tow truck driver for help.<br>**C. Call a paratransit to pick you up and take you home.**<br>D. Call 911 for help. home.
18. You need to change the engine oil of your modified vehicle for wheelchair accessibility. However, car manufacturers refuse to change the oil because of the modification, and vehicle modification companies do not provide the oil change service. How do you get your oil changed?<br>A. Do not change the oil and hope for the best.<br>**B. Call local mechanics to ask if they will perform the service for you.**<br>C. Leave bad reviews about the car manufacturer on the internet.<br>D. Sue the car manufacturer.
19. You are a power wheelchair user and notice that your wheelchair battery is low while traveling with your family. You do not have a charger with you. What do you do?<br>A. Ignore the low battery and continue traveling.<br>B. Put your chair into manual mode and have your family push you.<br>**C. Only move your chair when necessary and have the chair powered off when sitting.**<br>D. Go back home to charge the wheelchair.
20. You are a manual wheelchair user. You are not sure how much the public transportation system is accommodating for people with disabilities in your area. You have errands to run, how do you handle finding out about public transportation accessibility?<br>**A. Call the transportation customer service hotline, make inquiries, ask relevant questions, and plan a route.**<br>B. Ask a stranger who looks like a local and see if they can offer any guidance.

C. Call a friend in the area who can pick you up and attend to your needs
D. Decide to just stay home.

21. You are a power wheelchair user, and you are on a work trip in a new city. All your coworkers are taking a taxi service to a dinner. You are unsure if the taxi service has accessible taxis. How would you handle this?
A. Offer to arrange the taxi service so that you can ensure it is wheelchair accessible.
B. Ask the travel plan coordinator to get an accessible taxi for you.
C. Tell your coworkers that you have to ride separately and arrange your own transportation.
D. Decide that you cannot go.

22. You are a power wheelchair user. You hit a hole or crack in the sidewalk causing you to be thrown out of your wheelchair. Your wheelchair remains upright, but you require assistance to get back in. What do you do?
A. Attempt to get yourself back in the wheelchair, which requires a great feat of strength
B. Lay in the middle of the sidewalk and loudly ask for help and hope a passersby will come to your aid
C. Call 911
D. Accept your fate and just lay there thinking about life

23. You are a manual wheelchair user. While waiting for a paratransit vehicle you go inside a convenience store. They call you but your phone was muted, and you missed the call and they leave without you. What do you do?
A. Call the paratransit help number to see if the driver can come back
B. Ask a stranger for a lift to your destination.
C. Call a family member or friend to come pick you up
D. Start rolling as fast as you can in the direction that you think the driver went in hopes of catching up to them

24. You are a power wheelchair user getting on a bus. Multiple people are blocking the designated seating areas for people with disabilities. What do you do?
A. Maneuver your wheelchair into the middle of the aisle and just ride to your destination.
B. Politely ask the other passengers to move so you can ride the bus comfortably.
C. Ask the bus driver to tell the passengers to move so you can access the designated seats.
D. Roll over the feet of the passengers sitting where you should be able to sit while staring at them.

25. You are a manual wheelchair user crossing a crosswalk. A car rolls up and waits at the stop sign but the driver impatiently honks at you. What do you do?
A. Continue going across the crosswalk at a steady pace. No need to get involved with the driver.
B. Ask another pedestrian for help crossing the crosswalk at a faster pace.
C. Make note of the car's license plate number so you can report the driver to the authorities.
D. Move extra slowly across the street and stare down the driver.

26. You are a manual wheelchair user who has been out and about for many hours today. Your hands and arms are growing tired, and blisters are forming. What do you do to make the rest of your day more comfortable?
A. Push through the pain and buy a pair of wheelchair hand gloves to give your hands some protection later.
B. Ask another pedestrian to push you and give your hands a break.
C. Stop pushing and call a friend for a ride.
D. Find a nice shady spot and take a nap.

27. You are a power wheelchair user and while grocery shopping you notice that the joystick controller on your wheelchair is not operating correctly, causing delays and difficulty moving. What should you do?<br>A. Put the chair into manual mode and get assistance being pushed.<br>B. Contact the service department of the wheelchair vendor for guidance.<br>**C. Try to reset the system by turning it on and off.**<br>D. Keep using the chair and hope no other issues arise.
28. You are a power wheelchair user. You are riding the bus and get off 2 stops before your stop but don't realize until the bus has already put the ramp back in. What do you do?<br>**A. Ask the driver to put the ramp back out so you can get back on the bus.**<br>B. Drive to your destination on the sidewalk.<br>C. Call a paratransit to take you to your destination.<br>D. Wait for the next bus and get back on to get to your stop.
29. You are a power wheelchair user. Your current chair is three years old, but you need a replacement due to wear and tear. Insurance will only cover a new chair every 5 years. What do you do?<br>A. Fix up your wheelchair yourself on your own dime.<br>B. Ask another wheelchair user if you can borrow one of their old ones.<br>**C. Call your insurance provider and attempt to negotiate an early replacement.**<br>D. Continue using the wheelchair and hope that it can keep going.
30. You are a power wheelchair user visiting a museum. The museum has an accessible ramp but there is a delivery truck blocking the ramp. What do you do?<br>A. Wait for the delivery truck to leave.<br>**B. Ask museum staff is there is an alternate accessible entrance.**<br>**C. Ask the delivery driver to move the truck.**<br>D. Decide not to visit the museum and go home.
31. You are a power wheelchair user and must use public transport to get around. Someone insults you on the bus. How do you respond?<br>**A. Ignore them.**<br>**B. Address the person politely and educate them about people with disabilities and the issues they face when using transportation.**<br>C. Use your phone and start recording them for video evidence.<br>D. Get off the bus and wait to get on the next bus to continue your route.
32. You are a manual wheelchair user and just got accepted to the college of your dreams! It is a small college, and they lack accessible entrances to some of the buildings on campus. What do you do?<br>A. Attend your second or third choice school because they offer more accessibility.<br>B. Enquire about online options for classes that you would be unable to attend in person.<br>**C. Appeal to the college dean in hopes that they will make their campus more accessible as dictated by the ADA.**<br>D. Attend a school that is all online, so accessibility is not an issue.
33. You are a wheelchair user but ambulatory for a short distance. You are using the bathroom at a restaurant and while there is a larger stall designed for wheelchair access, it is too narrow to allow for a comfortable entrance and exit with your wheelchair. What do you do?<br>A. Still maneuver your wheelchair through the door, making multiple-point turns to get in and out.<br>B. Get up and leave your wheelchair out of the stall and hope no one takes it.

C. **Ask the restaurant manager if there is another bathroom, such as the employee bathroom, that has a bigger doorway.**
D. Leave your wheelchair at the table and walk to the restroom.

34. You are a manual wheelchair user using the underground metro system to get around town. As you get into the station, you realize that the turnstiles that lead to the platform are not accessible and there is no accessible gate. What do you do?

A. Attempt to slide through the turnstile and pull your wheelchair behind you.
B. Ask another passenger if they can help you through the turnstile.
C. Ask a metro employee if there is an accessible entrance you can use to get on the subway.
D. Condemn public transportation and give up.

35. You are a manual wheelchair user planning a road trip with friends. As you begin to discuss the logistics of driving across the country, you realize that you require extra space for all of your equipment and your wheelchair which will limit cargo space. What do you do?

A. Decide it will be too much stress and not go on the trip.
B. Ask your friends if they would be okay with bringing less stuff so you can travel comfortably.
C. Call the car rental agency and spend more to find a vehicle that can accommodate your wheelchair along with your luggage.
D. Tell your friends that you won't be able to go on the trip due to a distant uncle's wedding.

36. You are a power wheelchair user attending a concert on the other side of the city to see your favorite band. You are unsure if there will be accessible public transportation. What can you do?

A. Research local public transportation options and buy a weekend bus pass.
B. Ask a friend or family member if they could take you to the concert.
C. Call the customer service line for your city's transportation service and make sure that they have wheelchair-accessible buses that you can access.
D. Decide that it is too stressful and skip the concert.

37. You are a manual wheelchair user but ambulatory for short distances. You work in a tall office building and the elevator in your building has stopped working. It will take at least one day to fix. What do you do?

A. Tell your boss that you can work from home until the elevator is fixed.
B. Ask one of your coworkers to assist you up the stairs.
C. Call the building manager and insist that the elevator be fixed overnight as it prevents you from working and properly accessing the building.
D. Quit your job and find someplace more accessible to work.

38. You are a manual wheelchair user. It begins to snow, and your wheelchair becomes difficult to maneuver through the open streets. What do you do?

A. Put on some warm gloves and continue to push through the snow.
B. Find a shop to go in and wait for the snow to slow down.
C. Ask a passerby to help push you through the snow.
D. Travel to a bus stop and take a bus instead of pushing yourself.

39. You are a manual wheelchair user on your way to a business meeting downtown. Rain begins to pour from the sky, threatening your professional attire. What do you do?

A. Open your umbrella and try to propel your wheelchair and hold the umbrella.
B. Ask another pedestrian if they could help push you to your destination while you stay dry underneath the umbrella.
C. Wait out the rain and call the people you are meeting with and ask to reschedule.

D. Accept that you'll show up to the meeting soaking wet and go as fast as you can.

40. You are a power wheelchair user. You want to visit a famous landmark, but you are unsure if it will be wheelchair accessible. What can you do?

A. Go online and see if the landmark has an accessibility page for you to research and plan ahead.
B. Ask your family members to scope out the landmark ahead of time so you are sure you can attend.
C. Call the landmarks management office and ask about their accessibility options and potential accommodations.
D. Determine that visiting the landmark is not worth the hassle.

41. You are a power wheelchair user making your way down the sidewalk, but many shops and restaurants have placed signs that are blocking the sidewalk, and limit your ability to get around. What do you do?

A. Try to move the signs yourself and continue on the sidewalk.
B. Ask another pedestrian to help you maneuver around the signs.
C. Ask an employee of one of the stores to move the sign and explain that they are obstacles for people using wheelchairs.
D. Return later that day with some spray paint and show the restaurants and shops who's boss.

42. You are a power wheelchair user moving along the sidewalk when a large group of people are moving the other way towards you. What do you do?

A. Slow down and try to make the pass as easy as possible for everyone.
B. Stop moving and allow the pedestrians to move by you first.
C. Ask one of the pedestrians to help you get by them so that they can continue walking together.
D. Speed up your pace while moving into the center of the sidewalk and hope they move over otherwise you will bulldoze them.

43. You are a power wheelchair user. You are planning to go to the beach with your family, but you are unsure if the beach has accessibility. What do you do?

A. Research your destination to see if there are accessible walkways or provided mobility assistance.
B. Ask a family member to help transport you to the beach and back.
C. Call a local mobility service and inquire about specialty beach wheelchairs that have wheels designed for use in sand.
D. Skip out on the beach trip and suggest another vacation spot.

44. You are a power wheelchair user trying to find a parking space in the city, but all of the accessible spaces are blocked by outdoor seating for restaurants. What do you do?

A. Submit a complaint to the city and request new accessible spots or removal of the blockages.
B. Order takeout and ask them to bring it out to the car for you.
C. Park but take up two spots to ensure enough space.
D. Decide to give up and just go home.

45. You are a power wheelchair user. You are shopping at the grocery store and need assistance reaching an item on the top shelf. How do you get the item?

A. Stand up and attempt to reach the desired ingredient from the top shelf.
B. Ask another customer to get it for you.
C. Find the store manager and request shopping assistance.
D. Give up and find something different to eat.

46. You are a power wheelchair user and are in a new city. You make your way to the bus stop but are completely confused by the signage and schedule. What do you do?

A. Look for other directional cues near the bus stop or on their website to orient you.

B. Ask a fellow local waiting for the bus if they can assist you.<br>**C. Call the transportation authorities to complain/ask for directions.**<br>D. Give up and vow to never return to this city ever again.
47. You are a manual wheelchair user. You are in an unfamiliar area of the city and are trying to get home, but you are unsure where to go or how to access public transportation. What do you do?<br>A. Persevere and find the best route on your own<br>B. Look around at local shops or popular destinations to see if they have public transport information.<br>**C. Use a map on your phone to try and decipher to best route from what you are able to find.**<br>**D. Call the transportation authority for instructions and better guidance.**
48. You are a power wheelchair user, and you arrive at a shop and observe that the sidewalks are not wheelchair accessible. How do you get into the store?<br>A. Launch a formal complaint against the company saying that they are not in regulation of the ADA requirements and should be shut down<br>**B. Ask a stranger for assistance to see if they can assist you getting over the curb and into their store front**<br>C. Submit a complaint that states that this business discriminates against disabled people<br>D. Contact the construction company who built your friends store front and demand that they shave down the sidewalk immediately
49. You are a manual wheelchair user. You notice that your city has curb cuts and accessible sidewalks but there are some with a steep slope that are very difficult to navigate. What do you do?<br>A. See if your insurance can upgrade your wheelchair to provide mobility assistance to you when you encounter these situations.<br>B. Contact your local lawmakers and bring this gap in policy to their attention.<br>C. Start a petition to make the city sidewalks more accessible<br>**D. Get assistance when going down the steeper sidewalks.**
50. You are a power wheelchair user. You arrive at an establishment and the accessible parking spaces are taken by able-bodied persons. How do you handle this?<br>A. Call a tow truck to move the vehicle.<br>**B. Take down the license plate number and take a picture to report the violation.**<br>C. Go in to the establishment and see if they can call the person to get them to move the vehicle.<br>D. Wait for them to come back out, ask them to move their vehicle and explain the importance of leaving handicap accessible parking spots for those who need them.

Note. Most frequently selected answers as most appropriate for the scenarios were marked with bold. Each scenario has a most appropriate answer but there are two for scenarios 22, 30, 31, 47.
